# Gender-based violence in Latin America (Ecuador and Argentina): current state and challenges in the development of psychoeducational materials

**DOI:** 10.1007/s44202-022-00060-4

**Published:** 2022-12-27

**Authors:** Joselyn Pispira, Jazmín Cevasco, María Luisa Silva

**Affiliations:** 1 University of Buenos Aires (Argentina), Lavalle 2353, Buenos Aires, Argentina; 2 National Scientific and Technical Research Council (Argentina), Lavalle 2353, Buenos Aires, Argentina; 3 Interdisciplinary Centre for Research in Psychology and Experimental Mathematics (CIIPME), Buenos Aires, Argentina

**Keywords:** Gender-based violence, Psychoeducation, Prevention, Discourse comprehension

## Abstract

Gender-based violence (GBV) is a complex social and public health problem, which represents a human rights violation. Globally, GBV tends to occur in intimate partner relationships. Latin American countries report high rates of this violence. Despite their social and historical differences, Ecuador and Argentina are among Latin countries that aim to dismantle patriarchy. Developing psychoeducation materials is one way in which communities can recognize and prevent GBV. Psycholinguistics can provide useful tools to facilitate learning about this social issue: prior studies suggest that promoting the establishment of discourse connections, the generation of emotion inferences and the emotional involvement of the comprehender facilitate written and spoken discourse comprehension. The aim of this commentary article is to present an overview of the current incidence of GBV in Ecuador and Argentina, and to highlight the contributions that preliminary research on discourse comprehension can make to facilitate learning about GBV prevention strategies. Finally, we will discuss possible research guidelines and future directions. We expect that this manuscript will contribute to highlighting the importance of promoting social awareness of GBV and, therefore, the crucial role of the design and implementation of scientifically based interventions.

## Introduction

Gender-based violence (GBV) is a complex social problem, which involves the systematic use and abuse of power and control against women, regardless of their ethnicity, nationality, social status, migratory status, sexual orientation, education [1, 2]. Consequences on women’s physical and mental health include injuries, chronic pain, depression, suicidal thoughts, gastrointestinal and gynaecological problems [3–5]. In consequence, gender violence is recognized as a complex public health problem and a human rights violation [6]. One of the most fruitful resources to prevent GVB behaviors is the design and implementation of public policies that prevent them. Therefore, it is important to examine the effectiveness of programmes that focus on promoting the change of attitudes and beliefs related to GBV, by mainly informing the public about victims’ rights and support networks [7, 8]. Previous studies that have assessed the effect of the implementation of these programmes have tended to focus on the United States, Spain and some European and African countries [9–13]. Few studies have examined GBV in Latin America, and specifically in countries such as Argentina and Ecuador. In addition, prior studies about GBV programmes have not tended to examine the role that psychoeducation materials play in the facilitation of learning about GBV prevention [14–17]. Given that these countries exhibit high rates of GBV, it is critically important to design effective interdisciplinary interventions. The aim of this article is to provide an overview of the current state of GBV in Ecuador and Argentina, and to highlight the contributions that research on discourse comprehension can make to the facilitation of learning about GBV prevention strategies. Finally, we discuss possible future directions.

## Gender-based violence in Ecuador and Argentina

GBV takes many forms (physical, psychological, verbal and sexual), and can occur in public (*work, streets, schools*) or private spheres (*family* and *intimate partner relationships,* [2, 18]. Yet, the World Health Organization [6] highlights, GBV is really prevalent in intimate partner relationships: 27% of women aged 15–49 have suffered physical and/or sexual violence by an intimate partner at some point in their lives. In Latin America and the Caribbean, this rate reaches 25% [19].

This article focuses on GBV in Ecuador and Argentina. Even though these countries do not share frontiers, and have social and historical differences, they both exhibit similar cultural patriarchal beliefs (‘*men represent power and strength in the public and private spheres*’, ‘*women should focus on caregiving and motherhood*’, [20]), which include a high social tolerance to GBV [21, 22]. These countries also share economic inequalities resulting from neoliberal policies, which lead to overexploitation of women in the workplace [23]. In relation to the fight against GBV, Argentina leads a feminist movement *“Ni Una Menos”* (*“Not One [Woman] Less”*) organized by female journalists, which campaigns against GBV [24]. The social need for these movements became clear, when more than 200.000 people gathered in a massive protest against GBV in front of the National Congress of Argentina in 2015. This massive protest promoted that other countries developed similar movements [25]. Among them, Ecuador developed a national movement: ¨*Vivas nos queremos*¨ (‘*We want to be alive*’), which organizes protests against the high rates of femicides [26]. These movements against GBV highlight the need for international research collaboration. Taking this into consideration, our paper focuses on the current state of GBV in Ecuador and Argentina, and proposes future directions for both countries.

In Ecuador, the National Survey on Family Relationships and Gender-Based Violence Against Women conducted by the National Institute of Statistic and Censuses [27] indicated that, during their lifetime, 56.9% of Ecuadorian women have suffered psychological, 35.4%, physical, and 32.7% sexual violence. The most frequent GBV behavior tends to occur in intimate relationships: 42.8% of women have experienced violence by their intimate partners. The Integrated Security Service ECU 911 [28], reported 103.516 emergency calls about violence against women or other members of the family in 2021. Fifty-five percent was related to psychological violence, 32.7% to domestic violence, 13% to physical violence, and 0.1% to sexual violence. These reports occurred during the COVID 19 pandemic. This was a very difficult time for women, given that confinement measures increased the risk of violence, and made it harder for them to have access to support services [29, 30]. In consequence, the observed decrease of 7.8% in emergency calls in 2020 (102.799 calls) in comparison to 2019 (111.472 calls), has been proposed to be related to fear that women experienced in relation to their abusive partners being at home [3]. Consistent with this idea, emergency calls increased again in 2021 [28].

In Argentina, the National Institute of Statistic and Censuses [31] reported 576.360 cases between 2013 and 2018. The most frequent form of violence was psychological (86%), followed by physical (56.3%), patrimonial (16.8%), symbolic (20.1%), and sexual (7.5%). Fifty-two percent of the cases reported multiple types of violence.

In Ecuador, women tend to experience GBV by their current (43%), or former intimate partners (39.1%). In Argentina, a helpline for victims of GBV provided by the Ministry of Women, Gender, and Diversity of Argentina reported 113.340 calls in 2021 [32]. Ninety-three percent were related to intimate relationships: 49% involved a former partner and 34% a current partner. Ninety-five percent of these calls involved psychological, 67% physical, and 14% sexual violence. Recently, the MMGyD [33] conducted a survey on the prevalence of GBV against women for the first time. Results indicated that 45% of Argentine women who are or have been in an intimate relationship have experienced some type of GBV in their lifetime. Sixty-four percent of the aggressors were former partners, and 25% current partners. The most frequent type of violence was psychological (23%), followed by patrimonial (23%), and sexual (18%). This survey also indicated that, although GBV affects all women, the percentage is higher among young women who have had no access to higher education.

Women who experience GBV also face barriers to legal assistance: lack of awareness of legal rights and options, fear of retribution from their aggressor, etc. [34, 35]. In Ecuador, 88% of women who have suffered psychological violence (e.g.,: they answered ‘*yes*’ to the question: ‘*Has your partner threatened you with a weapon?*’) never reported it to the authorities, and 53% told an acquaintance [27]. In Argentina, 31.9% of women who experienced physical violence by their partner and/or former partner did not reach out to anyone in their social network or any institutions, 35.5% sought support from their social network and from an institution, 23.2% reached out to their support networks, and 11.5% only to an institution [33].

These data suggest that intimate partner relationships can involve vulnerability and risk for women, and even end in murder [36]. Globally, a woman or girl is killed by a member of her family every 11 min. Fifty-eight percent of these crimes are committed by their intimate partners [37]. These crimes are legally considered *femicides,* given that they point to a sense of ownership over women's bodies and lives [38]. The Gender Equality Observatory for Latin America and the Caribbean [39], which is part of the Economic Commission for Latin America and the Caribbean (CEPAL), reported that 4091 women were victims of femicide. In 2021, 251 femicides were reported in Argentina [40], and 197 in Ecuador. In 2022, 272 femicides have been reported between January and November [41, 42]. These numbers highlight the magnitude of GBV. In fact, though a *femicide* is the most crude expression of violence against women, it really is the final stage of a continuum of violence [38]. In relation to this, it has been proposed that men who commit femicide are part of a culture that promotes dominance and control over women. These beliefs have been identified in interviews with perpetrators, who have stated: “*She was my rock, she was mine. I did not think so much about her*” ([43], p. 11) or *"If you’re not mine, you can´t belong to anyone else”* ([44], p. 252). Reducing women to the status of objects (i.e., “*mine*”, “*belong to*”) enables sexist men to feel entitled to control and manipulate them [45]. Examples of these patriarchal beliefs have been identified by prior studies: *"women should not work, they should be stay-at-home moms”, "women are weak, they need a man to protect them"* or “*only women know how take care of children*” [21, 46], “*women should dress in a way that does not provoke men*” [27].

To sum up, the high GBV rates suggest that it is part of a cultural system that reproduces gender stereotypes and prejudices [20, 22]. Although both Ecuador and Argentina have recently implemented laws to punish GBV [34, 38, 47], they still need to develop public policies to prevent it. In consequence, it is key that the scientific community gets involved in research that contributes to the prevention of this social issue [48].

## How can the prevention of GBV be promoted?

GBV prevention programmes tend to be based on a Social Norms Feedback-Based approach, and to focus on improving participants' knowledge about violence in intimate partner relationships, identifying risk factors, building support networks, and providing information about how to report abusers [8, 9, 49–51]. Their implementation tends to include group discussions, the presentation of audiovisual materials, role playing, reflective questions about personal and collective experiences, etc. [52–54]. Even though these interventions have been successful in promoting the identification of early signs of violence and beliefs that justify it, both in Ecuador and Argentina they have not focused on the facilitation of participants' learning from psychoeducation materials on GBV [14, 17, 55]. This gap is important, given that these materials are key to promote that participants learn about how to identify whether themselves, their relatives or friends are in a in relationship that could involve GBV, and how to intervene early [51]

The development of psychoeducational materials that facilitate learning about the prevention of GBV can be challenging, because they need to be based on effective prior research [17, 56]. In relation to this, prior studies on the facilitation of learning from narrative and expository discourse have not tended to focus on spontaneously produced materials on social issues such as GBV, but rather on research-created materials on other topics [14, 15, 17, 57]. In consequence, we do not know if their conclusions apply to the comprehension of materials on this social issue. Prior studies suggest that the establishment of causal connections is key to facilitate the construction of a coherent representation of written and spoken discourse. The role of the establishment of these connections has been examined by the *Causal Network Model* [58, 59]. This model proposes that a causal connection exists between two statements, when it can be proposed that the one that is considered cause is *temporarily prior* (that is, it occurs before its consequence), *operative* (it is active when the consequence occurs) and *necessary in the described circumstances* for the consequence to occur [59, 60]. That is, when it can be proposed that if the cause had not occurred, then the consequence would not have occurred. For example, in *The call of the wild* [61], the authors describes:

1. Every hour was filled with shock and surprise.
2. He had been flung into the heart of things primordial.

In this case, it can be proposed that statement 2 causes statement 1, because the protagonist’s ‘*being flung into the heart of things primordial*’ occurs prior to his ‘*every hour being filled with shock and surprise*’, is in operation when it does, and is necessary for it to happen. Prior studies suggest that events with many causal connections are recalled more often [56, 62–65], rated as more important [59], more often included in summarization protocols [66], more often identified as introducing a topic shift [67], and retrieved more quickly [68] than events with few connections in the comprehension of written and spoken discourse. In turn, the generation of *emotion inferences* have been proposed to enrich the mental representation that comprehenders construct of the text [69–74]. These elaborative inferences involve establishing a causal connection between an event and the psychological reaction that it is expected to trigger, using our prior knowledge on emotions [71, 72, 75–77]. Prior studies suggest that they can be generated when the comprehender establishes causal connections among statements that represent *goals* (the protagonist's or speaker’s desires that motivate subsequent actions), *attempts* (actions that are carried out in order to achieve the goal) and *outcomes* (changes of state resulting from these attempts or from other events [77–79]. For example, in a fragment of the fable Rapunzel (analyzed by [78]), the author describes:

‘*One day a woman longed for rampion that she could see through her window*’, (GOAL)

‘*In the twilight of the evening, her husband clambered down over the wall, hastily*

*clutched a handful of rampion*’ (ATTEMPT)

‘*and took it to her’* (OUTCOME).

In this example, we can infer that the woman experiences HAPPINESS. We expect comprehenders’ to generate this inference, given that the protagonist succeeded in her attempt to obtain a goal [70, 79]*.* The processing of discourse and the generation of emotion inferences have also been found to facilitate that comprehenders experience emotions [72, 80, 81]. Prior studies suggest that these emotions promote that the comprehender engages with the material [80, 82]. Other investigations have highlighted that discourse comprehension can be facilitated when comprehenders are asked to answer *elaborative questions* (such as ‘*why do you think that the statement you just read makes sense*?’, [83]). These questions are expected to promote the integration of the comprehender’s prior knowledge with the new information provided by the text or discourse [84–88]. Considering the importance of these factors for discourse comprehension [15, 70, 83, 89–92], current work in our lab is examining their role in the facilitation of the comprehension of GBV psychoeducation materials. In order to explore this topic, we asked a group of Ecuadorian female students to listen or read a 10-min interview with an Ecuadorian survivor of GBV focused on prevention [17]. We examined the comprehension by these students, considering the described high tolerance of GBV in this country, and the increasing number of GBV cases [27]. In order to explore the role of the establishment of discourse connections, we identified the number of causal connections of each of the speaker’s statements (following the described criteria proposed by the *Causal Network Model*). This allowed us to identify which statements had a high number of them, which have been proposed to represent the *main ideas* of the material [93, 94]. In order to establish which of these *main ideas* also promoted the *generation of emotion inferences*, we identified statements that represented outcomes of attempts to obtain goals, following prior studies [70, 77, 79]. These analyses allowed us to identify 5 *main emotional statements* in the interview (that is, 5 statements that had high causal connectivity and promoted the generation of an emotion inference). For example:

Interviewee: “I left my aggressor, and never came back”.

This statement has a high number of causal connections in the interview (it is connected to 9 statements), and promotes the generation of an emotion inference (*happiness*). In order to examine the role of the described variables, students were asked to listen to or to read the interview. After listening or reading, they were presented with the *5 main emotional ideas* in written, and were randomly assigned to answer elaborative questions in one of three conditions: *focused on the identification of main ideas* (participants had to decide whether each of the 5 main emotional statements represented a main idea: *Do you consider that the statement you just read represents one of the main ideas of the material? Yes/No. Why?*), *focused on the identification of the speaker's emotions* (participants had to decide whether they considered that the speaker experienced an emotion when they produced each statement: *Do you consider that the speaker experienced any emotions when she said this? Yes/No. Why?*)*,* or *focused on the identification of the comprehender's emotions* (after reading each statement, participants had to decide whether they experienced any emotions in relation to them: *Do you experience any emotions in relation to this statement? Yes/No. Why?*)*.* Results indicated that students who answered elaborative questions *focused on their emotions* included statements with a high number of causal connections in their answers to a greater extent than those that performed this task *focused on the speaker’s emotions*. This preliminary finding suggests that promoting that students focus on their emotions facilitates that they activate and elaborate on those statements that have high causal connectivity to a greater extent than asking them to focus on the speaker's emotions. This is important, given that these statements play a key role in the construction of a coherent representation of the material [15, 16, 95]. In consequence, promoting that students get emotionally involved appears to facilitate that they elaborate on statements that are key to comprehend and learn from the material. This finding converges with recent studies, which suggest that establishing a high number of discourse connections [67, 70, 81, 96], getting emotionally involved with the material [72, 80], and performing elaborative question answering tasks facilitate discourse comprehension [85, 97]. It also expands these studies, given that it focuses on the interplay between these variables, and on the comprehension of written and spoken spontaneous discourse on the prevention of GBV. Among possible limitations of this preliminary study, we can highlight that it examined female college students from only one Latin American country: Ecuador. It would be, thus, interesting that future studies compared the role of these variables in the comprehension of students from other countries, such as Argentina. This would allow us to explore whether we would observe the same results (this is possible, given that prior research has already suggested that the establishment of causal connections and the generation of emotion inferences play a role in written and spoken discourse comprehension by college students from this country [67, 70, 89]. It is also possible that results would differ, given that there are cultural and educational differences between these two countries [98]. Future studies should also examine the role of these variables in the comprehension of *male students*. Results could differ from ours, given that it has been suggested that prior knowledge and emotions that men experience when reading about GBV differ from those of women [99]. Another limitation of this study is that we did not examine the role of *emotional intelligence.* This is important, given that this variable has been observed to have a role in participants’ ability to identify emotions in others (that is, to generate *emotion inferences*), in themselves (to experience *comprehenders’ emotions*), and in the implementation of strategies to reduce or intensify and emotional responses (*emotion regulation*, [100]). In relation to this variable, prior studies also suggest that there are gender differences: men appear to have more difficulty experiencing, labeling and controlling their emotions reactions than women [101, 102]. In consequence, this variable could play an important role in listening and reading comprehension about GBV prevention [103]. Another limitation is that we did not examine the role of prior knowledge and beliefs about GBV [74].

In relation to *practical applications* of these findings, we can propose that it can be useful for teachers, psychologists, and social workers, to consider selecting, designing and presenting psychoeducational materials that facilitate that students and clients establish discourse connections, and that promote that they get emotionally involved with the text or discourse. In consequence, it would be useful, when selecting and designing materials on GBV prevention, to identify *main ideas* and *emotional statements,* and highlight them or include them as part of handouts and powerpoint presentations.

## Conclusions

The aim of this work was to describe the current prevalence of GBV in Ecuador and Argentina, and to present contributions that preliminary research on discourse comprehension can make to the design of evidence based psychoeducation materials on the prevention of GBV.

With this aim, we presented data that highlights the prevalence of GBV in Ecuador and Argentina, mainly in intimate relationships. It should be noted that recent reports indicate a dramatic increase in the number of femicides in both countries. Considering that GBV represents a public health problem with immediate and long-term individual and social consequences, we believe that the design and selection of evidence based psychoeducation materials could be a key to eradicate the social matrix of GBV.

In order to design effective interventions and programmes, the involvement of the scientific community is crucial. Our preliminary research suggests that the establishment of a high number of discourse connections, and the presentation of elaborative questions promoting comprehenders’ emotional involvement facilitate the comprehension and learning from psychoeducational materials on GBV. These findings converge with studies that have indicated that discourse comprehension can promote the activation of intense emotions [74]. They also expand prior studies, given that they highlight that we can apply psycholinguistic knowledge to promote active learning from materials on social issues, such as GBV prevention [14, 15, 17]. Our findings also contribute to the promotion of the development and implementation of interventions that are evidence based.

## Future directions and recommendations

Taking into account these preliminary findings, we can propose that it can be useful that future studies continue examining the effect of the design, selection and presentation of materials that promote the comprehension of topics related to the prevention of GBV. These materials should promote the establishment of discourse connections (not only *causal,* but also *adversative, additive* [15, 67, 104]), and facilitate that participants get actively engaged in learning about this topic (for example, through the presentation of *elaborative question answering tasks*). It would also be interesting that future studies examined whether participants’ active learning can be facilitated by asking participants to perform other tasks that promote comprehenders’ oriented processes, such as *self explanation* and *note-taking* [14, 94]. Other investigations could also examine the interplay between the establishment of discourse connections and the performance of elaborative question answering tasks in the comprehension of *refutation texts* [105]. These texts explicitly present misconceptions (this can include *prior incorrect beliefs about GBV*), refute them and present the correct information [106].

In conclusion, we hope that this work will promote new directions in educational practices and research, which can contribute to the promotion of the design of materials on socially relevant topics.

## Declarations

### Data availability

Data sharing not applicable – no new data generated.
